# Precambrian Lunar Volcanic Protolife

**DOI:** 10.3390/ijms10062681

**Published:** 2009-06-11

**Authors:** Jack Green

**Affiliations:** Department of Geology, California State University, Long Beach, California, 90840, USA; E-Mail: jgreen3@csulb.edu; Tel. +1-562-985-4198; Fax: +1-562-985-8638

**Keywords:** fumaroles, protolife, caldera, energy sources, polymerase chain reaction, lunar sensors

## Abstract

Five representative terrestrial analogs of lunar craters are detailed relevant to Precambrian fumarolic activity. Fumarolic fluids contain the ingredients for protolife. Energy sources to derive formaldehyde, amino acids and related compounds could be by flow charging, charge separation and volcanic shock. With no photodecomposition in shadow, most fumarolic fluids at 40 K would persist over geologically long time periods. Relatively abundant tungsten would permit creation of critical enzymes, Fischer-Tropsch reactions could form polycyclic aromatic hydrocarbons and soluble volcanic polyphosphates would enable assembly of nucleic acids. Fumarolic stimuli factors are described. Orbital and lander sensors specific to protolife exploration including combined Raman/laser-induced breakdown spectrocsopy are evaluated.

## 1. Introduction

This paper is limited to volcanic processes and is tied in to terrestrial analogs modified by lunar environmental constraints. As with many geological systems, the fumarolic environment represents an open system—the broken test tube, the defective blast furnace. Moreover, pure reagents are virtually unknown in nature and variability is the rule. Tidal and gravity effects may have played a major role in intensifying Hadean-Archean lunar volcanism [1] as they have on Io. The vertical component of the K_2_ Love number of 0.0266 (as a vector pointing to the earth) shows that the moon is more rigid than the earth with its K_2_ Love number of 0.35 [2]. Thus lunar body tides on the moon are as much as 10 cm compared with as much as 50 cm for the earth. However, what is often overlooked is the tidal response of fluids in the moon, which would be greater than the gravitative effect of the moon on the earth [3]; this may explain the occurrence of endogenic lunar transients reported in historic time. Finally, the moon exhibits no plate dislocations. On earth, Benioff-style plate tectonics since 1.3 billion years has apparently resulted in suture-controlled volcanism on rifts and in subduction zones. The moon, in contrast, exhibits regional, not suture controlled volcanism. Of course, major and minor meteorite impacts have overprinted all lunar volcanic terrains.

The author believes lunar protolife was created on the early Precambrian. As defined by the International Commission on Stratigraphy (of the International Union of Geological Sciences), the Precambrian time period is honored but actually has no rank. It is simply the time period before the Cambrian. This time period is formally broken down into the Proterozoic (0.540 – 2.5 billion years), the Archean (2.5 – 4.0 billion years) and the Hadean (4.0 – 4.6 billion years). For the beginning of lunar protolife, this paper considers the time period since lunar differentiation through the Hadean and into the early Archean. This period is based on the assumption that the earth-moon system differentiated at about the same time because the oldest terrestrial rocks based on zircon ages is 4.404 billion years old and the oldest lunar highland rocks are 4.46 billion years old. Accepting the possibility of lunar protolife, the writer does not speculate on the following issues: (1) the timing of when protolife first appeared on the moon, (2) the timing of when protolife first appeared on the earth and (3) any progression of lunar protolife beyond self-replicating DNA. The author defines lunar protolife as the assemblage of volcanic compounds via lipids, catalysts, linkages and templates through organic entities capable of self-replication and progression.

Finally the goals of this paper are clear. The author details favorability criteria of fumaroles for protolife at every step in the progression of amino acids to DNA. Energy sources for protolife were available and fumarolic fluids could be retained in lunar shadow. Certainly a major goal was to highlight the remarkable combination of circumstances that prevailed in the early Precambrian environment – the soluble polyphosphates, the tungsten catalysts, the clay and pyrite templates and the eH, pH and temperature changes within meters.

## 2. Scope

The threefold sections of this paper includes: (A) target sites for lunar protolife exploration, (B) evolution of protolife from Hadean-Archean lunar fumarolic fluids and (C) selected remote sensors for evidence of protolife. Only a brief review of tidal and gravity factors intensifying lunar volcanism is covered. The major part of section A deals with potential fumarolic sites in shadow or under dust. Several terrestrial analogs of these sites are presented. In section B, the discussion excludes exotic source compounds such as silanes, but instead emphasizes fumarolic fluids found on earth. Modifications to terrestrial volcanic fluids are based on reasonable extrapolations to the Hadean and Archean. Energy sources, protolife building blocks, Fischer-Tropsch reactions, enzymes, condensation, encapsulation mechanisms, biofilms and unique aspects of lunar environments are covered. A stimulus concept for creating protolife in lunar fumaroles is detailed. In section C, selected remote sensing instruments are described with emphasis on their ability to penetrate dust and to detect and record organic compounds.

## 3. Section A - Target Sites for Lunar Protolife Exploration

Protolife loci would logically occur in volcanic areas of the moon because fumarolic fluids and sublimates contain the ingredients for protolife. For the purposes of this paper, protolife includes specified compounds evolving to and including self-replicating DNA. Shadowed volcanic sites are prime protolife exploration sites and include the following:

1. Volcanic vent interiors, especially breached volcanoes within calderas.
2. Volcanic domes, especially dimpled domes and others within calderas.
3. Dark spots at fracture intersections often with small summit vents.
4. Polar shadowed zones, including those with intermittent sunlight on caldera floors.
5. Aligned craterlets in grabens, on “loops” or on tangents to calderas.
6. Fractures in shadow, especially at intersections on fractured caldera floors some coincident with documented endogenic transients.

Also, the younger the feature, the lesser thickness of impact and pyroclastic dust cover. High slope angles would also contribute to a lesser thickness of dust.

**Figure 1.**
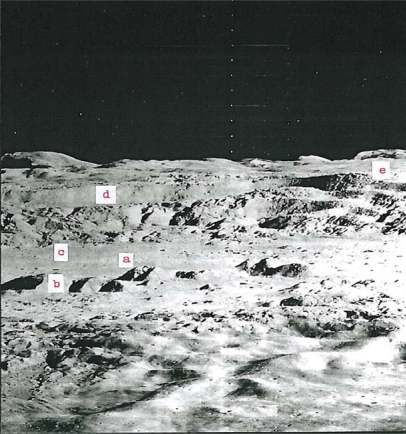
Protolife target site #1 – Breached volcano and associated features. Interior of Copernicus (97) km diameter) showing (a) possible breached volcano produced by a directed volcanic blast, (b) multiple volcano-like structures on crater floor similar to multiple volcanoes on the floor of the Tengger caldera in west Java, (c) sinuous leveed channels similar to pyroxene andesite lava levees in the Gedeh caldera, west Java, (d) horizontal terraces of different ages similar to terraces in the Fernandina caldera, Galapagos Islands, and (e) apparent low dip angles of rim rocks as in calderas on earth. Photo of Lunar Orbiter II, Frame 162 H_3_ courtesy of the National Space Science Data Center and principal investigator Mr. L.J. Kosofsky.

Regarding target site #1, we note the lunar-terrestrial analog of the Aniakchak caldera in Alaska with that of Regiomontanus on the moon; diameters in kilometers are 9.7 and 108, respectively. A well-formed volcano-like structure occurs on the floor of Schrödinger near the South Pole imaged in target site #4. The breached mountain of the multiple central mountain complex in Copernicus (97 km in diameter) (Figure 1) has been discussed by Green [4] and is believed to be caused by a lateral volcanic blast. The association of many geological factors leads to the presumption that this breached mountain in Copernicus is a volcano in a caldera. These factors are shown in Figure 1. The breached volcano-like mountain is similar to Mount St. Helens or Bezymianny and would be a prime target for exploration for protolife because a lateral blast would be younger than the volcano; a volcano would be younger than the host caldera and the caldera would generally be younger than the surrounding terrain.

The lunar crater Alphonsus is presumed to be a caldera. The crater contains a median ridge which extends into the polygonal wall of the adjacent crater Ptolemaeus. The ridge has a central mountain interpreted as a volcano, the summit of which exhibited activity in 1958. The “eruption” was spectroscopically documented by N. Kozyrev on November 3^rd^ as a verified lunar transient where carbon-bearing gases were released. The vent on this mountain would be an excellent target for protolife exploration. A terrestrial analog of Alphonsus is the Mokuaweoweo caldera in Hawaii. The caldera has a near median floor fissure which was active in 1940 and on which a large volcanic cone was formed. See oblique U.S. Navy photograph across Mokuaweoweo floor January 7, 1949.

**Figure 2.**
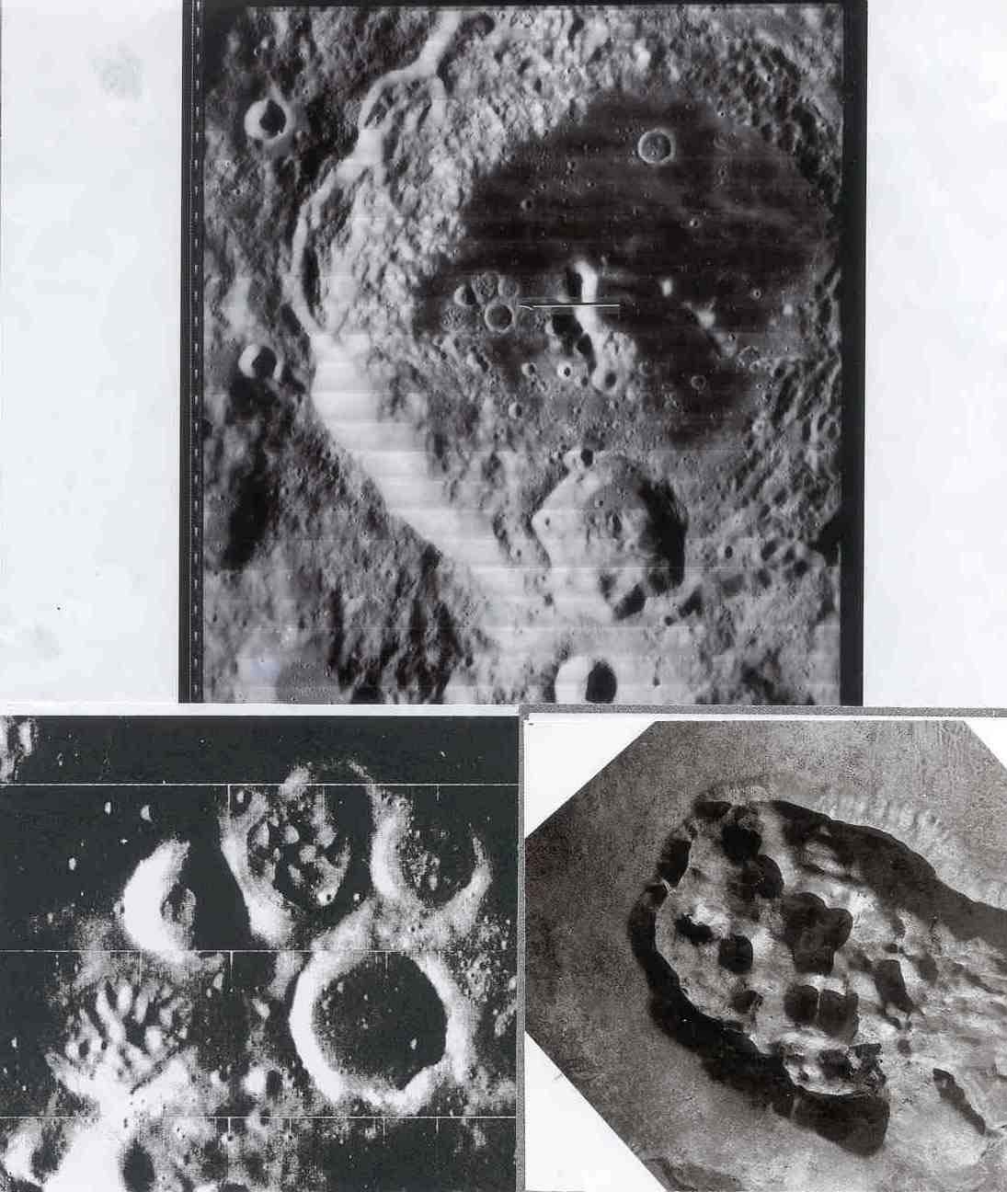
Protolife target site #2 – Volcanic domes. Domes in the Aitken crater. Aitken is located on the northern border of a major basin on the farside of the moon. The crater is about 150 km in diameter. An arrow in the upper Orbiter image points to a crater cluster within ramparts containing what appear to be volcanic domes. Another image of Aitken showing these domes is the Apollo 17 photograph AS17-151-23210. A high resolution Orbiter II image Frame 33 H shown on the bottom left is compared with a ramparted volcanic dome and crater cluster at Diamond Craters, (Central Crater Complex), Oregon. The approximate width of the dome cluster in Aitken is about 4.5 kilometers; that of Diamond Craters is about a kilometer. Hundreds of domal features occur on the moon, some with dimpled summits as in Euler, Alphonsus and near Hevelius and others within angular ramparts as in Barbier (60 km diameter). Shown is lunar Orbiter II photo (Frame 33H) courtesy of the National Space Science Data Center and principal investigator Mr. L.J. Kosofsky.

In volcanic site #2, we include domes, many of which have summit dimples. Some low albedo domes occur within the Copernicus “loop”. Those of the Marius Hills are well documented. An interesting cluster of domes occurs within a polygonal rampart in the Aitken crater. An analog of the domes in Aitken may be those of the Central Crater Complex of Diamond Craters, Oregon (Figure 2).

**Figure 3.**
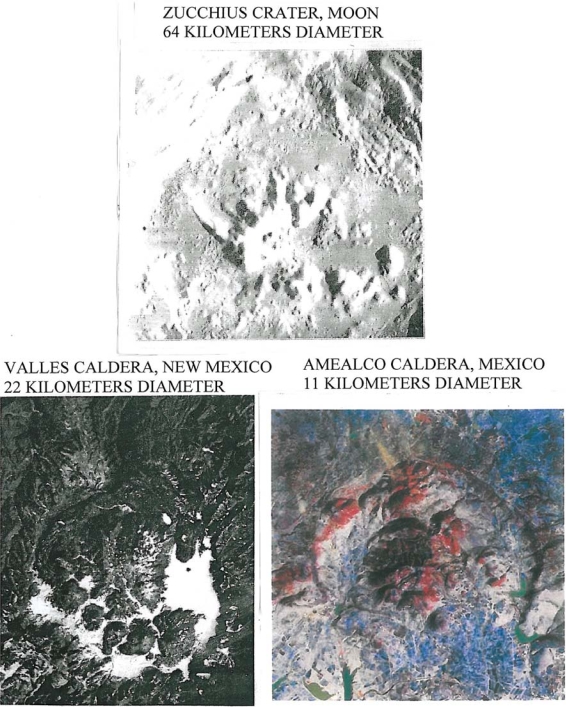
Comparison of arcuate central mountains in Zucchius (Smart-1 Imagery) with arcuate rhyolitic domes in the Valles caldera (NASA photo STS 062-100-195) and arcuate rhyolitic and trachyandesitic domes in the Amealco caldera, Mexico. Amealco imagery by CARDI (Mexico Digital Cartography) provided to CENAPRED (Centro Nacional de Prevención de Desastres). North to top for Zucchius and Amealco and bottom for Valles.

Another lunar crater with a well documented curving arc of interior domes is Zucchius; it is 64 km in diameter and 3.2 km deep. Although Zucchius, imaged by Smart-1 (European Space Agency) has been interpreted as an impact crater, the author believes it to be a caldera with internal volcanic domes similar to those in the Valles caldera, New Mexico and the Amealco caldera, Mexico. Note the arcuate pattern of the domes in Zucchius similar to the arcuate pattern of volcanic domes on fracture systems in the Valles and Amealco calderas in New Mexico and Mexico respectively (Figure 3). A detailed account of the genesis and petrography of the Amealco domes is given by Aguirre-Díaz and McDowell [5]. Volcanic domes on earth have been extensively studied for fumarolic activity (La Soufriére, Guadaloupe; Mount St. Helens, Oregon; Colima, Mexico; Lascar, Chile; Unzen, Japan; Redoubt Volcano, Alaska). Works by Howell Williams and Richard Stoiber are classics in the field.

Domal mountains are common in many tens of lunar craters and have been interpreted as caused by meteorite rebound. How to create multiple rebounds by impact requires unreasonable mechanisms. In Copernicus, for example, rebound would produce fayalitic olivine typical of deep-seated dunite. However, inspection of telescopic near infrared spectra shows the olivine to be forsteritic (Mg-rich) typical of spinifex mineralogy of komatiitic lavas.

**Figure 4.**
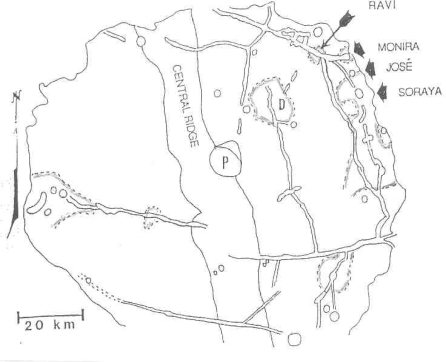
Protolife target site #3 – Dark spots on crater floors. Dashed lines enclose dark spots with small summit pits on the floor of Alphonsus. Some of the dark spots which may host buried fumarolic vents are named. Solid lines enclose craterlets except for “P” which is the central peak that was the source of carbon-bearing gases in a November 1958 lunar transient event recorded by N. Kozyrev in Russia. Dr. Nikolay A. Kozyrev, a Soviet astronomer, told the author in 1960 that he keenly wished to meet Dr. D. Alter, an American astronomer, who observed a haze in Alphonsus on October 26, 1956 that led to Kozyrev’s concentration on Alphonsus. Kozyrev (deceased February 27, 1983) was never able to travel to the United States. Dr. Dinsmore Alter died on September 20, 1968. For the purposes of this paper, the author calls the central ridge in Alphonsus, “Alter Ridge” and the central peak, “Kozyrev Peak.” Thus Kozyrev meets Alter. The map is based on 3.0-cm wavelength high resolution radar images and orbital photographs [6].

Regarding target site 3, dark spots with summit pits at fracture intersections in caldera-like craters such as Alphonsus may be younger than the crater floor fractures which in turn are younger than the host crater. Vents on dark spots, assumed to be associated with fumaroles, are common in many other craters on the moon. Note that Alphonsus is the site of a documented endogenic transient (Figure 4).

Target site #4 would be fumaroles in shadowed or intermittently shadowed polar crater floors at either lunar pole. Temperatures, in shadow, would be about 40 Kelvin resulting in the possible accumulation of fumarolic ices either disseminated or in isolated patches under unknown thicknesses of impact or pyroclastic dust. A sizeable literature exists for this type of target.

**Figure 5.**
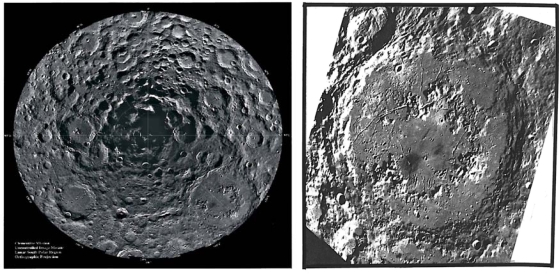
Protolife target site #4 – Polar shadowed zones. **(a) Lunar South Pole** Lunar South Pole region imaged by the Clementine probe of over 1500 UV-Visible images produced by the U.S. Geological Survey. Resolution is 200 meters. Crater in lower right-hand corner is Schrödinger (diameter is 320 km). Clementine mosaic is courtesy of the Naval Research Laboratory. **(b) Schrödinger Crater** The black spot in Schrödinger is believed to be one of the largest volcanoes on the moon. Note the summit pit in Schrödinger about 2 km in diameter and the interior partially concentric ring structure. Schrödinger is considered by this author to be a caldera.

In target site #4, volatiles produced by volcanic and volcano-tectonic processes would seek out polar cold traps. Volatiles generated by other mechanisms (icy comet impacts, solar wind, carbonaceous chondrite impacts) would likewise seek the shadowed crater floors of the poles. However, volatiles of any origin probably would not reach the poles, based on data by Hodges [7]. The migration time for a water molecule to reach the poles from the equator would require 5,000 saltations at 400 seconds each, requiring 2 × 10^6^ seconds of transit time. However, the photodissociation time of a water molecule on the moon is only 6 × 10^4^ seconds [8] as pointed out by Hodges. Therefore, water and likely most other volatiles could not reach the poles from the equator. Presumably, only volcanic volatiles generated within a radius of about 80 kilometers from the lunar poles could freeze out in shadow. Consequently, if polar craters are calderas, ices at the poles would be predominantly volcanic, accumulating *in situ* (Figure 5).

**Figure 6.**
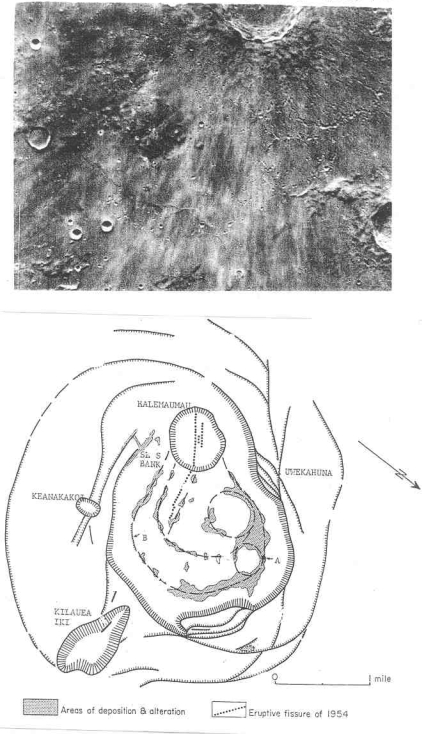
Protolife target site #5 – Crater flanks of Copernicus (top, north to right) exhibit well-defined “loop” patterns analogous to “loop” patterns [10] on the flank of Halemaumau, Hawaii (bottom) caused by inflation, deflation and migration of pockets of magma on the crater flank. The “loops” in Hawaii define fumaroles and craterlets (later covered with lava flows). The now extinct fumarole, Perret, is denoted by “A”. “B” is a fault scarp. The Copernicus “loop” also hosts a dark field of volcanic domes. Eratosthenes is in the lower right of top photo. Lunar photo is from Plate C 2439, Consolidated Lunar Atlas, Lunar and Planetary Laboratory, Houston.

Target site #5 would be fumaroles in fused craterlets or craterlets in chains, some of which are tangential to larger craters as at Müller or at Langrenus F, the latter near a well documented transient in Langrenus [9]. Admittedly fragmented meteorites can produce crater chains on impact but those that are in chains within a graben at the Rima Hyginus or tangent to craters must almost surely be volcanic. Also the Davy Crater chain of some 19 craterlets is curved. The most interesting fused craterlet and possible fumarole target area would be on the “loops” on the flank of Copernicus; a circumstance supporting the endogenic origin of this crater. “Loop” patterns often described as of an impact origin can be explained by inflation, deflation and migration of subjacent magma chambers on the flank of Copernicus. The craterlet chains on the “loops” are oriented on a regional tectonic fracture pattern trending northwest. Hadean-Archean tidal flexing would likely accentuate a lunar tectonic grid pattern because of the moon’s higher orbital eccentricity at that time and the greater radius of curvature of the lunar sphere. The chain alignments do not point back to the claimed impact locus in Copernicus. The craterlets show no lee or stoss gouge phenomena. An excellent analog of loops and ovals of fractures localizing craterlets and fumaroles [10] can be found on the flank of Halemaumau (Figure 6) in Kilauea.

**Figure 7.**
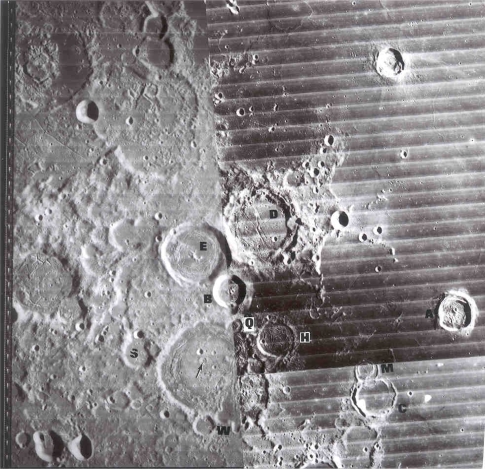
Protolife target site #6 – Ring fractures in calderas. Shadowed portions of fractures in the Lavoisier group of craters. Lavoisier has a diameter of 70 km. An arrow in this crater points to a small double ringed feature similar to craters C and M to the east with diameters of 35 and 18 km respectively. Other floor-fractured craters (H, E, and D) are shown as well as an unlettered crater on the western border. Crater D has been re-named “Von Braun”. Gaddis *et al.* [11] verify pyroclastic deposits in the Lavoisier area based on multispectral data from the Clementine mission. The spectral data resemble the lunar highlands with weak mafic bands and relatively high UV/VIS ratios. An impact origin of these craters resulting in volcanism is rejected. The Lavoisier area craters show features similar to over 40 calderas of *bona fide* volcanic origin on earth. Orbiter IV photos, Frame 189 H_2_, Frame 193 H_2_ and Frame 183 H_1_ courtesy of the National Space Science Data Center and principal investigator Mr. L.J. Kosofsky.

Finally, fractures which would be shadowed in the near subsurface constitute target site #6. Some fractures form polygonal craters such as Janssen, or are within craters as floor-fractured craters such as at Lavoisier (70 km diameter) where pyroclastic deposits [11] have been mapped (Figure 7). Schultz [12] has cataloged over 200 such craters, many with concentric inner fractures, and believes them to be volcanic craters produced by impact. The writer concurs with Ivanov and Melosh [13] that impact cannot produce volcanism.

## 4. Section B—Evolution of Protolife from Archean Lunar Fumarolic Fluids

As a preface to the formation of protolife in fumarolic systems on the moon, certain facts or concepts bear emphasizing:

1. Fumarolic fluids, prebiotic compounds, clay templates, catalysts and enzymes must react or “brew” within an enclosure such as in a double membrane micelle of a lipid or fatty acid.
2. Pure fatty acid micelles are only stable above pH 7, but if 20 mole percent of the equivalent-chain-length fatty alcohol is present, the resulting micelles are stable from pH 7 to pH 11 [14].
3. Only nanoconcentrations and nanocurrents are all that may be required to initiate protolife reactions in fumaroles.
4. Fumarolic fluids contain tungsten for the formation of certain catalysts, soluble polyphosphates for the formation of RNA or its precursors and boron compounds to stabilize ribose.
5. “Spark” genesis and Fischer-Tropsch catalysis for the generation of Archean prebiotic compounds may have operated contemporaneously in shadow on the moon.
6. Fumaroles with associated sublimates represent non-dispersed systems hosting multiple variables over distances of only a few meters.
7. Special physical attributes of fumaroles such as spatter can possibly result in a version of a polymerase chain reaction creating exponential replication of nucleotides.
8. Vapor pressures of fumarolic ices in shadow (40K) are so low that a centimeter thickness of these ices would persist for hundreds of millions to billions of years.

### 4.1. Energy Sources for Protolife

**Figure 8.**
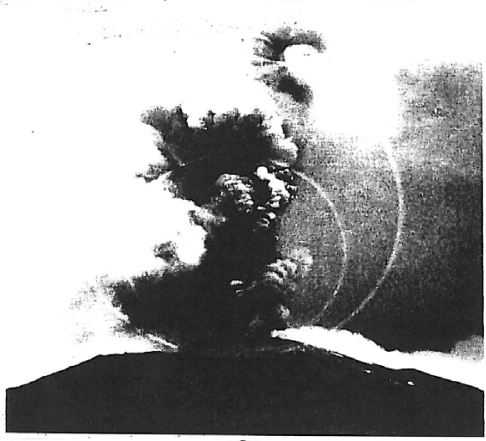
April 7, 1906 eruption of Vesuvius showing shock waves observed by Dr. F. Perret. Obviously, in 1906, cameras could not record this phenomenon. Perret drew them in. However, these waves are real.

There are several sources of energy available for the evolution of Archean lunar protolife in the form of sonic shock and electrical potentials. Sonic shock waves were first observed at Vesuvius on April 7, 1906 (Figure 8) by Dr. F. Perret. Since then, sonic wave overpressures have been measured up to 10 MPa at many volcanoes on earth (Shock Wave Research Center, Tohoku University, Sendai, Japan). Sonic shock in the Hadean time period could have been caused by volcanic eruptions, moonquakes, lighting and thunder. Thunder was cited as a cause for the production of Precambrian nucleic acids on earth by Dr. L. Orgel in 1973. Cavitation can produce shock waves in fumaroles at depth by rupture of silica, alunitic or clay cap rocks. Bassez [15] endorses a shock wave or a high pressure environment at depth in marine hydrothermal systems on earth to trigger prebiotic syntheses. Apolar molecules in high pressure environments would cause precursor molecules of CH_4_, H_2_, N_2_, H_2_S and CO_2_ with metal catalysts to form the building blocks of life. In 1959, El’piner and Sokolskaya [16] experimentally showed that shock waves in the presence of water, ammonia and carbon monoxide can produce prebiotic agents such as formaldehyde. Recently, meteorite impact modelling using rail gun instrumentation have shown that shock waves can polymerize amino acids to produce peptides [17]. Would volcanic shock do likewise?

Electrical potentials in fumaroles can result from flow charging [18] or triboelectricity [19]. Spectacular lightening effects have been documented for the Surtsey (Iceland) and Sakurajima (Japan) volcanic eruptions. A lightning bolt has some five billion joules of energy. Another source of electrical potential is called charge separation. Charge separation takes place in the freezing of dilute aqueous solutions [20] where cations or anions can be incorporated into the ice lattice resulting in a net charge. Freezing of ammonium thiocyanate, for example, under varying degrees of concentration, crystallization rate, nature of impurities, and pH, can generate up to 180 volts. Freezing of ammonium compounds, episodically repeated, could conceivably create amino acids and proteinoid microspheres with associated budding and fission in lunar fumaroles in shadow. Formaldehyde could also form in shadow “in the spark” as well as by methane-oxygen reactions. This 1959 quotation is by Dr. S.M. Miller relative to spark discharge in his original 1953 experiment involving methane, ammonia, hydrogen, and water vapor for the production of formaldehyde and other products at the First International Symposium on the Origin of Life on the Earth. (Moscow) Fumarolic thermal energy would be supplemented in the Hadean by now extinct radionuclides (Tc^129^, Sm^146^, Al ^26^, etc.).

### 4.2. Fumarolic Products

The author assumes an early a Hadean-Archean thermodynamically stable (non-reactive) anoxic and transient atmosphere of methane, ammonia, water and hydrogen [21] which would probably be concentrated in lunar topographic lows. In shadow the photolysis of methane would be minimized [22]. With increasing regional (non-suture controlled lunar volcanism), hydrogen would be lost and carbon dioxide, critical in prebiotic reactions, would be increased even in a non-aqueous medium [23] along with Archean sulfur and cyanogen-bearing gases. All these fluids—from volcanic vents, and endogenic lunar transients—except those frozen as ices or concentrated as heavy elements in the shadow of surface rocks—would have subsequently escaped, possibly sequentially as a function of their escape velocities and vapor pressures. Volcanic sublimates with the possible exception of cotonnite, P6Cl_2_, would also evaporate in lunar sunlight.

Major volcanic fluids include: ammonia, ammonium cyanide, carbon disulfide, carbon monoxide, carbon dioxide, carbonyl sulfide, chlorine, cyanogen, methane, nitrogen, sulfur, sulfur monochloride and water. Traces of ammonia plus ferrous iron oxides can form today at Mount Etna and Vesuvius by the reaction of the iron nitride siderazote (Fe_5_N_2_) with steam in high temperature fumaroles and on associated hot lavas. On the moon, larger amounts of ammonia would probably be produced in this way (in addition to magmatic ammonia) by more abundant iron nitrides in the reducing environments of Hadean volcanism. Inorganic methane is typically enriched in fluids from hydrothermal environments. Interestingly, the highest concentrations of methane on Mars [24] are centered over volcanic sites. Derivative compounds, possibly in trace amounts, include formaldehyde, ammonium chloride, ammonium thiocyanate (derived from ammonia and carbon disulfide,), carbon tetrachloride, hydrogen cyanide, hydrogen sulfide (derived from carbonyl sulfide), cyanogen chloride, cyanogen sulfide, sulfur dichloride, sulfur tetrachloride and polycyclic aromatic hydrocarbons (PAHs). Inorganic (?) ethane and ethene have also been reported in fumaroles in the Radkevich maar subsequent to the July 1973 eruption of Tiatia volcano in the Kuriles. The organic gases C_2_-C_3_ alkene-alkane pairs, isobutane, benzene, furane and thiophene are present in fumaroles at Lascar Volcano, Chile [25]. Boron, iodine and bromine sublimates common in volcanic emanations on earth are additional candidates. Quenched sulfur might be in the form of a translucent glass with dendrites of β sulfur. Sulfur is the most abundant volcanic sublimate on earth. Sulfur which dissolves in CO_2_ would tend to lower the vapor pressure of CO_2_. Hydrates of methane and carbon dioxide are also possible and by terrestrial analog could exist in the near surface on the moon. These hydrates on earth often sequester organic-rich compounds.

Regarding the abundance of water in the early Hadean of the moon, O’Hara [26] documents the feldspathic character, high incompatible element concentrations and lack of the Eu anomaly in the lunar highlands as a result of partial melting in the presence of water. The partial melting was followed by near surface fractionation and volatile losses.

Arguments against volcanic or endogenic water are that the majority of large lunar craters are not calderas and that the fugacity or partial pressure of oxygen in lunar melts is too low to permit hydrous environments. Most publications assume that the oxygen fugacity of terrestrial magmas is 10^−9^ atmospheres. Since oxygen is related to how hydrous a melt might become, the low fugacity of lunar rocks of 10^−13^ atmospheres has been a powerful argument against the possibility of “wet” rocks on the moon and hence a water resource. The basis for the claim that lunar rocks are dry is the measurement of fugacity of the areally abundant returned samples and not of lunar rocks collected at volcanic vent sites. Also note that Saal *et al.* [27] show that numerical modeling of diffusive degassing of the very low Ti volcanic glasses provides an estimate of a concentration of water of 745 ppm (higher than previously reported) with a minimum of 260 ppm at the 93% confidence level.

**Figure 9.**
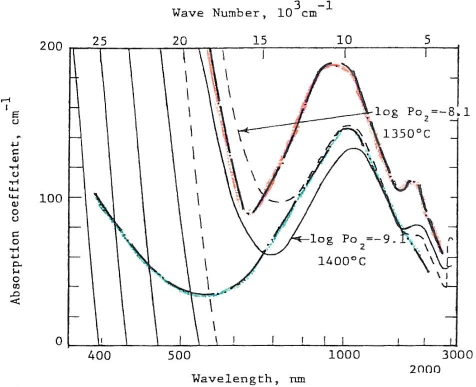
Unpolarized absorption spectra of “fire fountain” Apollo 17 orange and green glass from Apollo sample 74220.61 compared with synthetic glasses quenched at log Po_2_ at −9.1 (at 1350°C) and −8.1 (at 1400°C) modified after Mao *et al.* [28]. The synthetic glass of log Po_2_ = −9.1 is shown as a solid line; that of synthetic glass of log Po_2_ = −8.1 is shown as a dashed line. The absorption coefficient of the green (lunar) glass is superposed as a green line and is equivalent to a log Po_2_ of −8.1. The orange (lunar) glass is superposed as an orange line and is equivalent to a highly oxidized log Po_2_ significantly higher than −8.1. Terrestrial rocks have a partial pressure of oxygen of about 10^−9^ atmospheres. Hydrous melts (a function of the partial pressure of oxygen) were probable in the Archean on the moon.

Volcanic rocks at vent sites on earth are invariably more hydrothermally altered and “wetter” than distal lavas or ash flows. Are all lunar rocks low in oxygen fugacity? Mao *et al.* [28] performed optical absorbance studies of orange and green glass spherules from the Apollo 17 landing site. Optical absorbance is caused by crystal field and charge transfer processes in iron and titanium in these glasses. When these absorbance data are calibrated by structural parameters based on Fe^57^ Mossbauer resonance measurements, optical absorbance can be used to establish a scale of oxidation or fugacity. Optical absorbance data of synthetic glasses for which the fugacity is known are shown in Figure 9. Superposed are the optical absorbances of the orange and green glasses considered by most to be in the vicinity of a volcanic vent from the Apollo 17 site. The fugacities of these presumed volcanic vent (fire fountain) spherules are equal to or even greater than many terrestrial volcanic rocks. Work by Schreiber *et al.* [29], provides some justification for assuming water emission from lunar volcanic vents in the Archean. Volcanic vents on the moon may well represent loci for rocks and minerals of higher than 10^−13^ atmospheres of oxygen fugacity.

### 4.3. Building block production from primary fluids

Both electric discharge and Fischer-Tropsch catalysis were probably contemporaneous in creating prebiotic building blocks leading to the creation of RNA and DNA. Both mechanisms create lipids [30] of various types (including fatty acids, waxes, triglycerides and phospholipids) from aqueous solutions of formic and oxalic acids. Phospholipids are the principal component of cell membranes and are amphiphilic having hydrophobic tail groups and hydrophilic head groups forming a lipid bilayer [31]. Phosphorus is readily available as fumarolic soluble polyphosphates. Phospholipids fold upon themselves to form pockets or micelles in water under turbulent conditions [32]. Fumaroles almost invariably create turbulence. Protolife probably began within the semi-permeable double membraned lipid vesicles which facilitated reactions by minimizing external dilution effects. The degree to which the vesicles permit entrance or exit of compounds is a function of the length of their carbon chains (from 10 to 18).

In the 1953 Miller experiment, [33] an electric discharge was passed through a mixture of methane, ammonia, water and hydrogen in a reducing environment. This produced hydrogen cyanide, formaldehyde, acetaldehyde and minor amounts of unstable ribose, fatty acids and other compounds (Table 1). Johnson *et al.* [34], on re-running some of Miller’s original spark discharge samples, reports 22 amino acids and 5 amines in addition to those shown in Table 1.

Both hydrogen cyanide and formaldehyde would be stable in fumaroles in lunar shadow. Matthews [35] believes that the original polypeptides on earth were synthesized directly from HCN polymers and water. HCN polymers (as determined by Raman spectroscopy) were possibly present on earth in the 3.5 billion year old Apex cherts at Warrawoona in western Australia [36]. Both hydrogen cyanide and formaldehyde probably combined to initially form aminonitriles subsequently hydrolyzed to racemic hydroxyl amino acids. Amino acids can condense to form nucleosides, oligopeptides and oligonucleotides [37] possibly mediated in part by carbonyl sulfide or biofilms discussed below. Ferrocyanide, another important prebiotic agent, can ideally be formed in fumaroles by reacting hydrogen cyanide with ferrous iron to give Fe_2_Fe (CN)_6_. Purines (C_5_H_4_N_4_) and pyrimidines (C_4_H_4_N_2_) were formed in experiments subsequent to the Miller experiment. Segura and Navarro-Gonzáles [38] modified Miller’s experiment using CH_4_, H_2_, H_2_O, and N, producing acetylene (C_2_H_2_) and ethylene (C_2_H_4_). Purines and pyrimidines can also be formed by freezing dilute ammonium cyanide (NH_3_CN) [39]. Purines can be condensed by glycerol phosphate to make random chains of acyclic nucleic acids [40]. Sulfur compounds, not ingredients in the Miller experiment, are presumed to be present in fumarolic emanations; troilite (FeS) is common in lunar ferroan anorthosites and in the spherules brought back from the Apollo 14 and 17 missions. Reaction of H_2_S with elemental sulfur can produce polysulfides H_2_S_n_) leading to thiosuccinic aid [41]. Pyrimidine is the unsubstituted ring analog for three of the RNA and DNA bases: thymine, cytosine and uracil. It is a molecule of extreme importance for lunar protolife. In general, fumarolic shadow temperatures of 40K would retard hydrolysis and oxidation which degrade biomolecules and the organics noted above.

On the other hand, during the transient elevated temperatures when fumaroles were thermally active, ribose (with formose) can be formed in Fischer-Tropsch reactions by passing formaldehyde over hot kaolinite under low hydrogen cyanide concentrations. Although ribose is relatively unstable [42], its stability increases in the presence of borates—a common fumarolic compound—in the 80 to 100° C temperature range [43,44,45]. The author collected fumarolic incrustations at the Valley of Ten Thousand Smokes in Alaska, which had boron concentrations of 6700 ppm. Other sugar producing mechanisms involving carbon monoxide have been proposed by Aylward and Bofinger [46]. Fischer-Tropsch catalysis can create racemic basic and aromatic amino acids by passing fumarolic gases (NH_3_, CO and H_2_O) over hot (100–300° C) silicates in the presence of iron (or ilmenite?). From the Apollo 15 mission, native iron commonly overcoats pyroxene as 4 micron sublimation crystals [47,48]. Ilmenite is very abundant in mare basalts. The action of CO and H_2_ on hot mineral surfaces can also generate PAHs by Fischer-Tropsch catalysis [49]. PAHs have been identified in terrestrial volcanic ash by Podkletnov, [50].

### 4.4. Concentration and polymerization mechanisms

**Figure 10.**
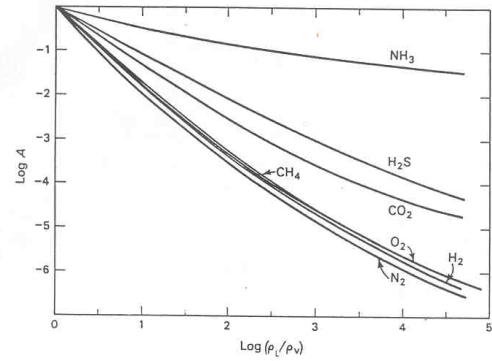
Fumarolic fluids may be enriched in ammonia. NH_3_/NH_4_ equilibria are governed by eH-pH relationships which vary widely in fumarolic/hot spring systems, the ammonium ion prevailing at higher temperatures and lower pH’s. In both the original plot and the one shown in this figure, the curves approximate straight lines which extrapolate from room temperatures up to the critical point value of unity (Log 0) at 374°C. Figure 10 shows that the solubility of various gases at low partial pressure is expressed as a function of the mass distribution coefficient “A” relative to the density of the gas in the liquid phase(ρ_L_) and vapor steam phase (ρ_V_):
$\text{A}=\frac{{\text{n}}_{\text{g}}^{\text{L}}/{\text{n}}_{{\text{H}}_{2}\text{O}}^{\text{L}}}{{\text{n}}_{\text{g}}^{\text{V}}/{\text{n}}_{{\text{H}}_{2}\text{O}}^{\text{V}}}$where n is the number of moles of gas (g) or water (H_2_O) in either the liquid (L) or vapor (V) phase (after Barnes [51]). The original plot by Ellis and Fyfe [52] (involving functions of “A” and log (ρ_L_) / ρ_V_) includes helium, but not methane, hydrogen sulfide and a more up-to-date curve of ammonia by Jones [53].

Concentration of all protolife components in fumarolic environments include several processes. Reflux in the vent enhanced possibly by magnified tidal cycles can concentrate fumarolic compounds analogous to telescoping and enrichment of ore deposits. According to Barnes [51], ammonia in geothermal fields is concentrated by steam/water fractionation (Figure 10). Apparently, the concentration of ammonia in geothermal fluids and fumaroles is a function of temperature and pH.

Although ammonia dissolved in water will increase the viscosity of water, the concentration of ammonia is too low in fumarolic fluids to significantly affect the viscosity. Magmatic ammonia is concentrated in several volcanic centers on earth such as White Island in the Bay of Plenty, New Zealand. Ammonia is an integral component of many cyanogen-based prebiotic compounds. Brandes *et al.* [54] conclude that ammonia on the Hadean-Archean earth would not be destroyed under hydrothermal conditions and may have facilitated the synthesis of amino acids and other essential biomolecules.

**Figure 11.**
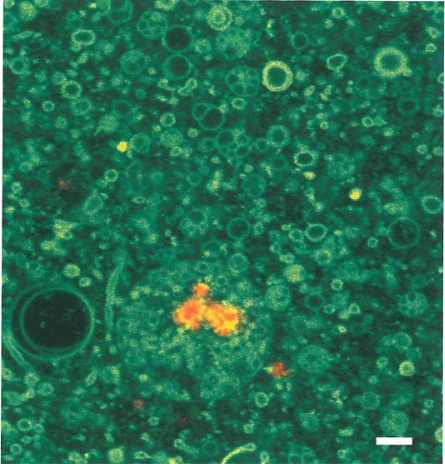
Red tagged RNA molecule bound to montmorillonite encapsulated in a lipid vesicle. Photo courtesy of Dr. M. Hanczyc, Howard Hughes Medical Institute, and Department of Molecular Biology, Mass. General Hospital, Boston, Mass. Bar scale is one micron.

Episodic freezing is another concentration mechanism and is fundamental to the origin of protolife in lunar shadow zones. Eutectic freezing of fumarolic fluids would result in ices of extremely low vapor pressure. If one cm thick at 40K, the ices would persist in shadow for hundreds of millions to billions of years. Freezing of ammonium cyanide and ammonium thiocyanate in the presence of formaldehyde creates proteinoid microspheres. (This is a different process than that used by Fox [55] to create these spheres). Ammonium thiocyanate can be produced by electric discharge in fumarolic gas mixtures containing hydrogen sulfide [56]. Amino acid polymerization on kaolinite and possibly amorphous opaline substrates [57] is another concentration mechanism [58]. Montmorillonite especially is a favored template [59,60] because of its expanding lattice and catalytic properties. These templates also prevent the decomposition of embedded amino acids [61]. Sheet structure mineral interlayers as occur in montmorillonite can concentrate potential reactants to about 10 molar [62]. Lipid micelles can also concentrate prebiotic compounds. In contact with montmorillonite, the acid nature of montmorillonite causes the micelle to enlarge to a vesicle exhibiting a pH gradient across the membrane interface. Figure 11 shows a lipid vesicle encapsulating a tagged RNA molecule bound to a montmorillonite substrate fragment within the vesicle [63,64]. The bar scale is one micron. Such encapsulation would permit enzymatic reactions to occur in semi-closed systems. Additional examples are detailed by Imai *et al.* [65] on the oligomerization of amino acids inside lipid vesicles in a hydrothermal setting and by Matsuno and Imai [66] on the encapsulation of glycine into oleic acid lipid vesicles at a hot/cold water interface. Such interfaces could be found in lunar fumaroles in shadow. Finally, concentration mechanisms would occur during wet/dry and hot/cold cycles in fumaroles and are discussed in a later section.

### 4.5. Chirality

A discussion of the chirality of prebiotic compounds is beyond the scope of this paper. There is a suggestion that an external magnetic field might bias chemical processes in favor of enantiomers—sugars on earth being dextrorotary (right handed) and biological amino acids being levorotary (left handed) [67]. One can speculate that the strong but short lived (100 million years?) magnetic field of the Archean moon may have affected the chirality of prebiotic compounds. On the other hand, some authors including Soai [68] and Heckl [69], believe that it was possible for purine-pyrimidine arrays to assemble in a specific chiral pattern on naturally occurring mineral surfaces. These purine and pyrimidine monolayers would then serve as templates for the assembly of higher-ordered polymers. Regardless, the great diversity of prebiotic conditions probably led to homochiral protolife from racemic initial conditions [70].

### 4.6. Precursors to RNA

Evolution of lunar protolife would require soluble polyphosphates which are found today in fumaroles at Mount Usu, Japan [71] as ortho-, pyro-, and tripolyphosphates. These phosphates have a high capacity for divalent ion base exchange [72]. Relationships between phosphorus and amino acids are discussed by Zhou *et al.* [73]. Purines, noted above, include adenine (C_5_H_3_N_4_NH_2_), produced by cooling ammonium cyanide (NH_4_CN). Purines can concentrate on pyrite surfaces affording protection from hydrolysis [74]. Adenine can react with the simple sugar, ribose (C_5_H_10_O_5_) to form adenosine (C_10_H_13_N_5_O_4_). In addition to its limited production in spark discharge experiments (which lacked metallic ion or double metal cyanide catalysis [75], ribose (with formose) can also form by Fischer Tropsch catalysis. Hydrothermal reactions including catalysis by green rust—a double layer iron hydroxide—may also play a role in enhanced production of ribose or ribose compounds [76,77]. Returning to adenosine, its reaction with water-soluble polyphosphates can produce adenosine monophosphate and then adenosine triphosphate—ATP (C_10_H_16_N_5_O_13_P_3_), a metabolic energy source. ATP may have been preceded by activated thioesters produced by simple fumarolic sulfur-bearing compounds by catalysis of metal sulfides, possibly troilite on the moon. Another energy source that may have catalyzed reactions to help form ATP would be voltages generated across thin walls of iron sulfide compartments separating different ion concentrations within the compartments and outside fluids. In the laboratory, Martin and Russell [78] have created cell-sized compartments in simulated iron sulfides which generated 600 millivolts between their thin walls. The voltage lasted several hours and is comparable to voltages across membranes of terrestrial living cells.

Regarding ATP, adenosine triphosphate assists in the polymerization of amino acids to proteins possibly by the addition or subtraction of phosphate groups: ATP to ADP to AMP. ADP is adenosine diphosphate and AMP is adenosine monophosphate. A considerable amount of energy is released when ATP splits back to ADP. ATP also promotes the formation of bilipid cell membranes.

The literature is burgeoning with other precursor mechanisms for the assembly of RNA, many of which are interrelated and all of which are amenable to a fumarolic environment. De Duve [32] espouses thioesters or threose nucleic acid (TNA) as a precursor to RNA. (Bonding a sulfur ion to a carbon-bearing entity called an acyl group yields a thioester). Jain *et al.* [79] appeal to a polycyclic aromatic hydrocarbon present in fumarolic fluids which is a planar tricyclic cationic molecule called proflavine. This small molecule acts as a “midwife” for assembling nucleotides into RNA at a rate 1,000 times faster than that in the absence of proflavine. Joyce [80] has reviewed other candidates for what may have preceded RNA including (1) pyranosyl-RNA (containing 4′, 2′-linked β- d-ribopyranosyl units), and (2) peptide nucleic acid (PNA), which can hybridize with DNA. PNA consists of a peptide-like backbone of glycine amino acid units with the bases attached through a methylene carbonyl group. Finally, Joyce admits that RNA-based life was possibly preceded by an early replicating evolving polymer that bore no resemblance to nucleic acids such as certain peptides. Was such replication realized by invasion of prebiotic viruses [81]? We don’t know.

The stage was now set for the appearance of an early version of RNA oligomers with its fumarolic components of purines, d-ribose and phosphates and with a mixture of mostly 2′,5′- and 3′,5′-linkages [56]. Some authors favor catalysis by montmorillonite [59] and dependency on zinc and magnesium [82] as necessary requirements for the appearance of RNA. RNA can act as a messenger, a catalyst and a progenitor of proteins (ribosomal RNA). Protein synthesis also may involve N-phosphoryl amino acids and the evolution of enzymes [83]. Ribosomal RNA has been shown to catalyze peptide chains during translation. Evolved complex ribosomes (“class 1”) may have appeared early in the RNA world with very high catalytic efficiency [84]. RNA adsorbed on clays, including montmorillonite, could possibly promote the enzymatic activity of RNA [85,86]. Some workers believe that life may have begun with a replicating enzyme of RNA [87]. Lincoln and Joyce [88] have created in the laboratory two RNA enzymes that copied one another and replicated 100 million fold in 30 hours. Others including Cairns-Smith [89] believe a simpler replicating system involving clay templates came first. Critical to this discussion is the range of environments assumed for lunar fumarolic protolife. Eutectic freezing of hydrogen cyanide in fumarolic fluids on earth may have been required to concentrate HCN to synthesize nucleic acid bases as well as purines and pyrimidines [90]. This reaction would be especially operative in lunar shadow. Also, Monnard [91] and Monnard and Szostak [92] have shown that nucleobase polymerization of RNA can take place in an ice matrix.

The single strand now self-replicating RNA according to Sukumaran [93] presumably evolved into the more stable double strand DNA (dsDNA). Then probably with a bridging medium of methyl-RNA [94], DNA appeared. DNA, the genetic blueprint for cell organization, consists of (1) a sugar, dioxyribose (C_5_H_10_O_4_), (2) a purine where thymine is exchanged for uracil and (3) an RNA “primer” [95].

### 4.7. Enzymes

Enzymes probably played a role in the origin of protolife prior and subsequent to RNA. For example, the proto-enzyme histidyl-histidine may have been fundamental during wet-dry cycles in fumaroles promoting peptide bond catalysis [96]. Tungsto-enzyme activation of amino acids as thioesters could have facilitated attachment of amino acids to the RNA molecule. Tungsto-enzymes may also post-date RNA. On earth, fumaroles can have up to a million-fold enrichment of tungsten over seawater [97]. These enzymes were a critical component for the evolution of Archaea on earth according to Kletzen and Adams [98].

### 4.8. Biofilms

Formaldehyde, sufficiently concentrated, is a frothing agent and promotes bubble formation with a colloidal iron sulfide membrane surface, i.e., a biofilm. Troilite is a common iron sulfide on the moon. In an aqueous medium FeS (troilite) + H_2_S → FeS_2_ (pyrite) + H_2_. This reaction has a large negative free energy of −41.9 kJ/mole assuring the viability of the reaction [99]. Colloidal iron sulfide as FeS_2_ would provide a positively charged biofilm or template to which polar organic molecules could attach and receive energy as surface metabolists. Ribosomal RNA has been extracted from present-day terrestrial biofilms from geothermal vents [100]. The presence of colloidal iron sulfide may increase the production of carbon dioxide by the oxidation of methane [101]. Troilite also promotes the formation of ammonia via the reduction of N_2_ with H_2_S as a reductant [102]. Colloidal iron nickel sulphides as biofilms and carbon monoxide in conjuction with common fumarolic fluids can activate amino acids to peptides [103]. Another mechanism to create iron sulfide biofilms is by reacting FeS, H_2_S and CO_2_ which form hydrogen and organic sulfur compounds, including methylthiols (CH_3_SH) and small amounts of carbon disulfide (CS_2_) and dimethyldisulfide [(CH_3_) _2_S_2_]. The latter effectively concentrates metals as metallo-thiols [104]. The overall reaction, rendering an aqueous surface hydrophobic, produces a biofilm coating of colloidal iron sulfide [105]. Methylthiol also reacts with carbon monoxide and water to make acetic acid, a prebiotic agent formed in the Miller experiment: CH_3_SH + CO + H_2_O → CH_3_COOH (acetic acid) + H_2_S. Hazen *et al.* [106] have studied mineral enhanced aspects of this equation. Recall that acetic acid and glycine condensations via several steps can lead to the creation of iron and magnesium-bearing porphyrins [107]. Methylthiol reactions [108] can also produce pyruvic acid, an energy source which unites key metabolic processes. The clay-coated bubbles of H_2_S in the Uzon fumaroles and hot springs of Kamchatka may provide a natural laboratory for the study of biofilms.

### 4.9. The stimulus concept for the origin of lunar protolife

All parameters vital to the evolution of protolife, both on the moon and the earth, are available in fumarolic systems within vertical and lateral distances of meters. These parameters include Eh, pH, pressure, temperature, wet/dry cycles, freeze/thaw cycles, and hot-cold fluid mixing involving glycine [66] (which can produce a 1000 fold increase in triglycine). Other parameters possibly stimulating the origin of lunar protolife (Table 2) include convection, agitation and reflux possibly by tidal cycling, electrical potentials and fluctuating clay environments [96,109]. Shock mechanisms may also play a role in protolife evolution. Low lunar gravity favors zeolite growth [110]. Zeolite “cages” can enclose and protect amino acid clusters. Two aspects of Table 2 are detailed below: A4. wet-dry cycles and J. lower gravity and surface pressure.

### 4.10. Wet-dry cycles

**Figure 12.**
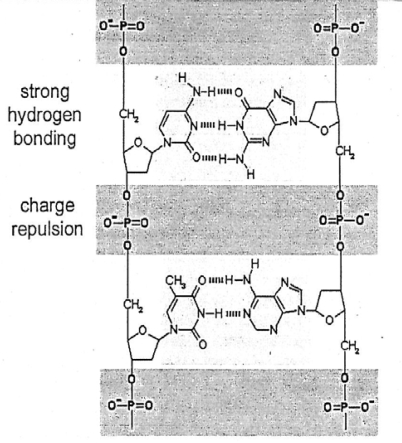
Idealized uncoiled helix of DNA. Reprinted from Lathe, R., “Fast tidal cycling and the origin of life”, *Icarus*, **2004**, *168*, 18–22, with permission from Elsevier.

Lathe [113] offers an insight into the origin of life on earth based on rapid tidal cycling when the rotation of the earth in the Hadean was presumably only a few hours in duration and when the moon was possibly much closer (200,000 km? [114,115]) Rapid tidal cycling would have produced enhanced advance and retreat of ocean water on coastal areas on earth. Wetting and drying phenomena resulting from this process may have stimulated the origin of life according to Lathe. Wetting would be associated with tidal advance, diluting concentrations of ocean salts. Drying would be associated with tidal retreat, and evaporation of ocean water with marked increases in concentrations of salts and polynucleotides. Under “dilute” seawater wetting environments, the opposing phosphate groups that separate each sugar-nucleotide monomer in DNA (Figure 12) would repel each other and the two strands of DNA would dissociate at low ionic strengths.

In drying environments characterized by high soluble cation concentrations, the phosphate charges would be neutralized and inter-strand hydrogen bonding would promote association of the two polymer strands favoring DNA replication. Bernal [116] notes that mineral surfaces could favor this association or polymerization process. Alignment of polymer precursors opposite a parental strand is favored by high precursor concentrations such as NaC1 during the drying phase on the terrestrial model. Copying by the DNA polynucleotide can only take place during the drying phase along with non-enzymatic polymerization through dehydration condensation. Replication would be exponential analogous to a polymerase chain reaction [113].

Lathe’s rapid tidal cycle concept can possibly be applied to the moon. He recognizes that his mechanism would be effective with DNA precursors such as dsRNA. Fumarolic monovalent ion concentrations such as ammonium or lithium enhance the folding and activity of catalytic RNA molecules [117]. Note that Lathe invokes relatively high monovalent concentrations of NaCl in terrestrial Hadean-Archean ocean water as the medium during the drying (ebb tide) phase for neutralizing the phosphate groups in DNA causing association. However, divalent ions in fumarolic fluids are concentrated on desiccation and are far more effective in stabilizing nucleic acid duplexes, including RNA. The high concentration of divalent ions would neutralize the phosphate charges in dsRNA promoting association of RNA strands favoring replication. These divalent ions such as Ca [118], Mg, Ba or Zn under periodic desiccating conditions would produce strong concentrations of organic molecules and enzymes which would minimize the possibility of hydrolysis [119]. Magnified tidal cycles on the Hadean-Archean moon could well enhance flow and ebb processes on the moon which are the hallmarks of fumarolic activity. Although there are many causes for such flow and ebb in terrestrial fumaroles today, outflow in Hadean-Archean lunar fumaroles—during apogee—could produce dilution by wetting. Inflow—during perigee—could produce salt concentration by drying. The respective dissociation and association effects could conceivably create a version of Lathe’s polymerase chain reaction by amplification of RNA molecules.

**Figure 13.**
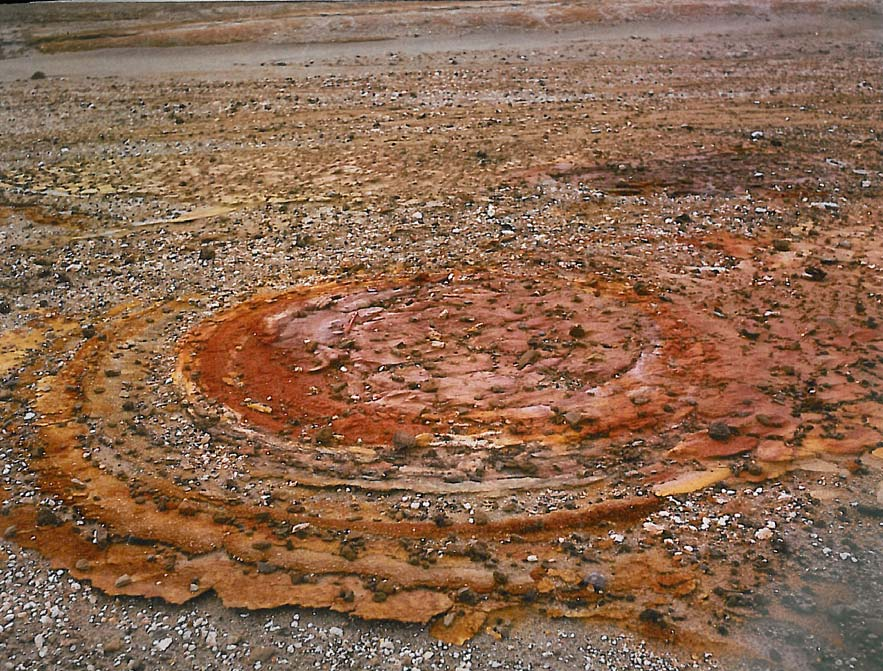
Desiccation and zoning features of an extinct fumarole in the Valley of Ten Thousand Smokes, Alaska. Width of fumarole is 2.5 meters. Photo by the author.

Regardless of this possibility, rapid tidal cycling of fluids in fumarolic vents would accentuate reflux phenomena in the vent. Such agitation can create phospholipids under favorable conditions [32]. The model this author favors most is (1) the concept of dripping or spattering of fumarolic mud or (2) aerosol contact onto hot or cold montmorillonitic or kaolinitic clays which would concentrate nucleotides, catalysts, enzymes and divalent cations. Stalactites of fumarolic sublimates and precipitates produced by dripping occur at the Tolbachik hydrothermal complex in Kamchatka. Drying cycles in Valley of Ten Thousand Smokes have produced the zonation and desiccation features illustrated in Figure 13. Note that wetting and drying of clay-rich vents have been shown to produce peptides of 15 to 20 amino acid chains [95,96].

**Figure 14.**
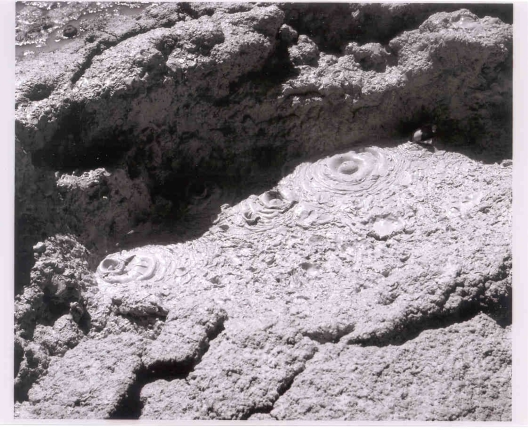
Spatter in boiling mud fumarole, Bumpass Hell, Lassen National Park, California. Image width is about 2.5 meters. Photo by the author.

Spatter in mud fumaroles (Figure 14) may have played a major role in both lunar and terrestrial prebiotic genesis. Spatter would invoke both fumarolic hot/cold and wet/dry cycles shown in Table 2. The distance fumarolic droplets could be thrown on the moon can be gauged from recorded throwout distances on earth. At the Helviti mud fumarole in Iceland in 1814, mud clots were thrown a distance of 9 meters which would correspond to 54 meters on the moon (based on a 45 degree ejection angle and an equatorial lunar gravity of 1.62 m/sec^2^). The terrestrial and lunar ejecta areas would be 254 and 9311 m^2^ respectively. A more violent mud pool eruption occurred in Kuirau Park in Rotorua in New Zealand. Here, on January 26, 2001, ejecta 0.1 m or less in diameter were thrown 100 m away. On the moon this would produce a blanket of 1.15 million square meters. Examples of active fumarole fields on earth include the Kawa Karaha in Java covering 250 × 80 m, and the Garbes in Djibouti, northeast Africa, 400 m long and 10’s of meters wide.

Thus, there would be a wide area in fumarole fields in shadow on the moon permitting dryout on both hot and cold surfaces with increasing distance from the vent. With spatter landing on cold clay, concentrations of pyrimidines including cytosine on drydown may result as has been postulated for a desiccating lagoonal environment in the Archean on earth [120,121]. Likewise, applicable to fumaroles in lunar shadow and the cold origin of protolife would be concentration of ammonium cyanide resulting from evaporation including spatter dryout on cold clay. Miyakawa *et al.* [90] have identified a wide variety of pyrimidines and purines in a frozen ammonium cyanide solution that had been held at −78° C for 27 years and cite its relevance to the cold origin of life on earth. Repeated freezing and thawing on the moon could have allowed the buildup of progressively larger chain molecules. Again, it is worth noting that fumarolic sites on the moon would be buried by unknown thicknesses of impact and volcanic ejecta.

### 4.11. Lower surface atmosphere pressure and lower gravity

Two uniquely lunar parameters favoring protolife in fumaroles are (1) low atmospheric surface pressure on the moon during the Hadean-Archean and (2) low lunar gravity. Low atmospheric pressure would reduce boiling points of prebiotic compounds such as formic acid (HCOOH), the most abundant product (255 × 10^5^ moles or 4%) in the original Miller experiment. Under hydrothermal conditions, formic acid is a precursor of lipids by thermocatalytic reactions [122]. Formic acid can also form in an aqueous solution by the reaction of troilite, hydrogen sulfide and carbon dioxide with a free energy of −11.7 kj/mole: FeS + H_2_S + CO_2_ → FeS_2_ + HCOOH. The boiling point of formic acid at 1 atm or 760 mm Hg is 100.7° C; at 120 mm Hg, the boiling point is 50° C – a temperature more hospitable for the evolution of life on earth. This pressure of 120 mm is equivalent to a depth of about 4 meters in a lunar fumarole where the shadow surface pressure today is essentially 0 mm (10^−15^ mm). Lower boiling points would also produce larger bubbles [123] and extend the vapor phase range of lunar prebiotic compounds enhancing reactivity.

**Figure 15.**
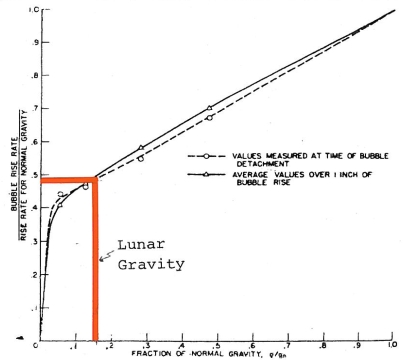
Slower rise rate of bubbles under low gravity modified after Usiskin and Siegel [124]. Under lunar gravity the rise rate of bubbles, all other factors held constant, will be about one half the rise rate of bubbles under earth gravity. Bubble diameters would also be about 1.7 times larger holding pressure constant.

Lower lunar gravity would result in a deeper nucleation of bubbles in the volcanic vent [124] enhancing caldera floor subsidence. Lower gravity would result in volcanoes and domes within calderas to be lower than caldera rims. A longer bubble entrained fumarolic column would increase biochemical reaction rates by a slower rise of bubbles (Figure 15). Reactivity would also be increased by convection (by smaller buoyancy forces) and fluidization [125].

## 5. Section C—Selected Remote Sensors for Evidence of Protolife

No attempt will be made to review the literature on lunar remote sensing. Some of the instruments on Chandrayaan-1 launched in September 2007 such as the Moon Mineralogy Mapper and sensors on the Chinese Orbiter Chang’e-l launched December 2006 and terminated in March 2009 are peripherally related to protolife detection. This in no way minimizes the excellence of proposed instrumentation for forthcoming missions. The objectives of remote sensing for protolife ideally require one meter resolution from orbit and centimeter resolution from surface sensors. Features of interest are (1) primary fluids from fumaroles H_2_O, CH_4_, CO_2_ and S and C compounds, (2) the presumed resulting building blocks for protolife, such as amino acids, ATP, RNA and DNA, and (3) intermediate products at the target sites noted in the text. Active sensors would need to be power limited (gated) as manned surface exploration evolves.

### 5.1. Orbital sensors

The addition of neutron spectroscopy to the CdZnTe gamma ray spectrometer [126] should permit detection of hydrogen from lunar orbit but not within a desirable one meter resolution. Detection of other elements O, Fe, Ti, A1, Si Ca, U, Th and K are not as germane to protolife detection. The Omega visible-infrared imaging spectrometer proposed for Martian exploration would be a valuable addition to lunar orbiters. LOLA (lunar orbiter laser altimeter) of the Lunar Reconnaissance Orbiter (LRO) launched on June 17, 2009 should provide lunar elevation data to less than one meter and horizontal positions to within 50 meters or less. These data should be useful in the characterization of the topography of dark spots at fracture intersections. The MoonLITE penetrometer could likely identify hydrothermal products in these mounds using X-ray fluorescence spectrometry. A similar instrument was launched on the Chang’e-1 probe. Regarding polar craters which may host volcanic ices, intermittent illumination of selected crater floors warmed to 220 K may create a transient tenuous atmosphere of CH_4_, H_2_S, CO, COS, HCI, CO_2_, and possibly H_2_O and which could be analyzed by near infrared spectrometry (NIMS) of SELENE or Chandrayaan-1. Prior to the 2009 impact of a polar crater by LCROSS (of the LRO mission), the Soviet LEND mission may detect water using epithermal neutrons. The impact plume proposed in the LCROSS mission at a polar crater could be analyzed by NIMS for fumarolic fluids similar to the NIMS analyses of Callisto and Ganymede moons of Jupiter. The possible identification of cyanogen in the LCROSS impact plume would support the CN_2_ spectrogram at Aristarchus by Kozyrev in 1969. In the Aristarchus region, lunar dawn during periods of maximum orbital flexing may accentuate release and detectionof Rn, Ar and protolife gases. These gases could possibly be identified by the Chang’e-1-type gamma/x ray spectrometer, NIMS and the neutral mass spectrometer of the LADEE mission. Microwave spectrometry and radar on the LEO mission as well as on LROC (LRO mission) could also be directed at verified lunar transient sites.

An orbiting sensor not yet instrumented would be an active (but gated) microwave laser to warm suspected accumulations of ices under dust followed by conventional lidar scanning such as a modified TIMS (thermal imaging mapping spectrometer). Other active orbiting sensors, not yet instrumented, could include an active orbiting linear accelerator for recording gamma backscatter from frozen volatiles, especially a 2.22 gamma signal specific to H-bearing ices or sublimates. An active orbiting CO_2_ laser (likewise gated) could be used for spot ablation of possible fumarole sites also followed by spectrometric vapor phase analysis.

An altitude sensor of merit relative to exploration for protolife is the P-band radar system which shows excellent surface penetration of unconsolidated material. The P-band synthetic aperture radar has been proposed for Mars exploration by Paillou *et al*. [127]. Their Figure 2 suggests that this instrument could be applied to the moon to possibly define fumaroles under pyroclastic and impact ejecta or to define thick ice under dust at the poles. Finally, an orbiting pole-to-pole bolometer would be a welcome addition to the orbiting sensor family.

### 5.2. Surface Sensors

Several sensors have been proposed for the detection and analysis of organic biomarkers on Mars. The most recent is the Urey Instrument for the 2013 Exomars Rover Mission described by Aubrey *et al.* [128]. The biomarkers targeted by the Urey Instrument include amino acids, nucleobases and amine degradation products. Measurements can be made of amino acid chirality which will serve to discriminate between abiotic and biological molecules. A lifeless setting would likely have equal amounts of right-handed and left-handed amino acids. Living organisms on earth assume the left-handed form. With temperature modification, this instrument could possibly be used in lunar exploration for protolife. Research related to the Urey instrument is ongoing in Kamchatka for characterization of biomarkers in hot spring and fumarolic environments by Toporski, Maule and Steele [129]. Analyses carried out in the field include highly sensitive LAL (Limulus amebocyte lysate) assays for the presence of bacterial cells, ATP measurements and microarray analyses.

The author proposes a simple instrument for detection of volatiles in polar craters. Recognizing that numerous polar craters have intermittent sunlight [130], many biogenic volatiles would be mobilized from 40 K in shadow to 220 K in intermittent polar sunlight [131]. A simple metal canister insulated on top and slotted on the bottom could be filled with honeycombed activated silica gel, carbon, or other gettering agents specific to possible volatiles. The unit could be dropped into polar craters with intermittent sunlight. Volatiles, if present, with boiling points below 220 K, would tend to be mobilized seeking out cold traps in the crater. After some months on the crater floor, the slots on the base of the canister could be electronically closed and the canisters recovered for laboratory analysis. Examples of possible volatiles with boiling points below 220 K would be methane, hydrogen sulfide, carbon monoxide, carbonyl sulfide, hydrogen chloride, carbon dioxide and possibly water. Boiling points at reduced pressures at around 220 K can be calculated using the Clausius Clapeyron equation based on molal heats of vaporization. At an arbitrary estimate of 0.1 mm of pressure at the ice interface on vaporization, the lowered boiling points are methane 55 K, hydrogen sulfide 115 K, carbon monoxide 41 K, carbonyl sulfide 116 K, hydrogen chloride 101 K, carbon dioxide 124 K and water 222 K.

**Figure 16.**
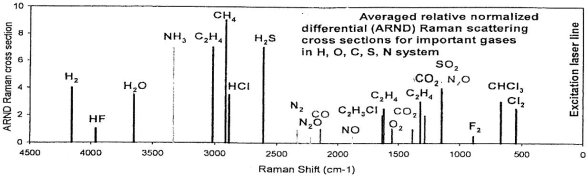
Raman spectrometry of important gases [134] many of which are candidate fumarolic gases.

Also, the 0.8 Kg Martian CHEMIN XRD/XRF instrument for *in situ* characterization of ices and hydrous minerals [132] appears to be an ideal robotic soft lander that could be adapted for lunar polar crater floor studies. That it can identify constituents of brines (F, C1 and S) is noteworthy. Raman spectroscopy is a desirable sensor for lunar exploration for protolife [133–135] with its capability to detect fumarolic gases (Figure 16). A new 440-nm laser diode module that can yield a high power output may result in the improvement of Raman spectroscopy according to a September 2004 report by Power Technology.

An example of its application would be in the investigation of the interior of the directed blast volcano (?) in Copernicus. Raman spectroscopy as applied to Martian soft lander or rover exploration [136] clearly identifies rock-making minerals and organics such as formaldehyde using a single laser pulse. Raman spectroscopy can easily identify pyrite which, if in the form of framboids [137], would possibly constitute a biomarker [138]. When combined with laser-induced breakdown spectroscopy [139], the two instruments would be ideal for selected protolife targets on the moon especially when both instruments (for Martian application) can be operated at a standoff distance of 10 meters. Laser ablation spectroscopy is excellent for mineral and rock analyses [140]. A miniaturized combined Raman and Laser-Induced Breakdown Spectrometer (LIBS) has been designed and built for the first time by the Dutch Organization for Applied Scientific Research—TNO (The Netherlands Organization). The unit was conceived as part of the MoonShot project [141] and a Development Model is available for accommodation on a lunar mission. The spectrometer is an excellent candidate as a lunar lander for the detection of protolife forms in shadowed fumarolic ices, sublimates and hydrothermal alteration products.

The realization that: (1) some elements in fumarolic ices or sublimates are high or relatively high in their neutron capture cross section [142], (2) boron compounds stabilize ribose, (3) chlorine may create ice brines and (4) sulfur as pyrite or troilite is a biofilm focuses our attention on nuclear spectroscopy. The neutron capture cross of boron is 767 barns, that of chlorine 32 and sulfur 0.53. Selenium is often associated with volcanic sulfur and has a cross section of 11.7 barns. Irradiating fumarolic ices or sublimates with thermalized neutrons produces a characteristic gamma backscatter the magnitude of which is proportional to the neutron capture cross section of the element. (In borehole geophysics, usually the interest is not in the capture cross section per element, but in capture cross section per unit volume called capture units). Nuclear spectroscopy, used routinely in well logging, could ideally be applied as a lunar surface drilling probe [142] at any of the protolife target types such as dark patches at fracture intersections in craters which may define subsurface fumaroles. The paper by Albats *et al.* [143] is specific to the application of nuclear spectroscopy as applied to lunar exploration but their emphasis is not on protolife. Possibly the 2015 lunar landers proposed by India and China will include nuclear spectroscopy.

The lunar space elevator [144], deemed speculative by this author, would probably be a late phase of lunar operations. A lunar space elevator is a flexible tethered structure connecting the lunar surface with counterweights located beyond the L1 or L2 Lagrangian points. Present day materials exist to construct a high strength ribbon or tether on which lunar robotic climbers could operate. There would be no weather, orbiting satellite collision or radiation hazards as would beset the earth space elevator. Pearson [144] points out that the lunar space elevator could be used to exploit lunar resources. This author awaits the reality of the lunar space elevator, but if it should be constructed, remote sensors which are very heavy such as a UV differential lidar (DIAL) could be attached to it. On the moon, this unit would weigh some 166 kg. Some sensors require much power such as a CO_2_ laser. Both of these sensors could be accommodated on a lunar space elevator at appropriate altitudes and could perform laser ablation breakdown spectroscopy. A more powerful alpha particle spectrometer mounted on a lunar space elevator could continuously monitor radon gas emanating from near Aristarchus and Kepler; a gamma ray spectrometer could monitor thorium enrichments near these two craters. In general, a much more thorough long-term study of transient phenomena could be made from remote sensing instruments on a lunar space elevator.

## 6. Conclusions

**Figure 17.**
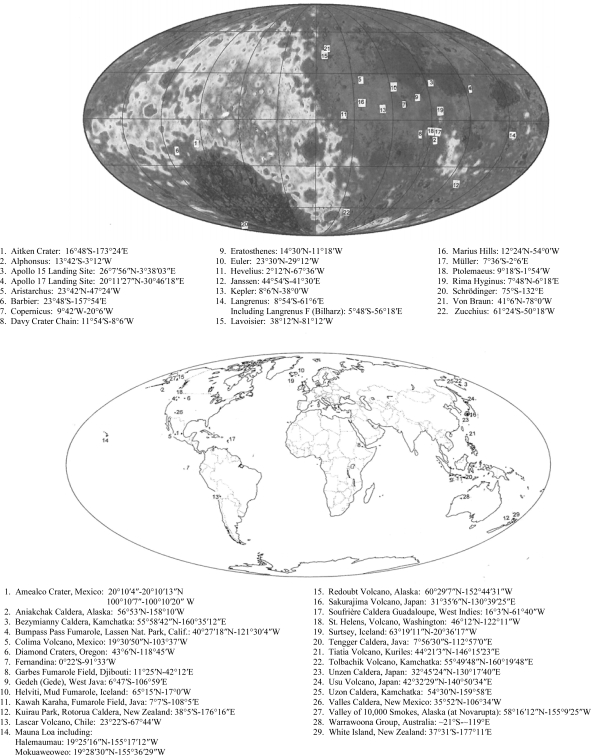
Craters and other features on the moon and earth cited in the text. Exact locations are given by latitude and longitude.

Many terrestrial analogs of lunar features have been presented to document the likelihood of early Precambrian fumarolic activity on the moon. Figure 17 illustrates the locations of the craters and other features cited in the text. The lunar base map is compiled from Clementine data and formatted in a Mollweide projection by Dr. Mark Wieczorek of the Institut de Physique Du Globe de Paris.

The moon in its early history, when tides and orbit were more extreme than now, may have been more environmentally favorable to the origin of protolife than on the earth. This is because: (1) shadowed sites on the moon would preserve critical fumarolic fluids as ices isolated from photodecomposition and (2) lower gravity and surface atmospheric pressures would enhance probabilities of fumarolic fluid reactivities. There are numerous factors promoting enhanced volcanism on the moon from the theoretical (tides and gravity) to the observational (lunar-terrestrial analogs). Lunar volcanic and volcano-tectonic features would have a meteoritic impact overprint. Favorable lunar volcanic sites for protolife exploration include: (1) shadowed vents, both in polar areas and in central mountains in presumed calderas, (2) in fractures, (3) within floor- fractured craters, (4) within dimpled domes and (5) at endogenic transient areas.

Once fumarolic activity was initiated at these sites, reactions stimulated by thermal and electrical energy and probably within amphiphilic micelles, presumably formed the building blocks of protolife (amino acids, purines and pyrimidines) from fumarolic fluids (H_2_O, CO_2_, NH_3_, HCHO, and S and C compounds). A chain of reactions probably involving (1) Fischer-Tropsch catalysis, (2) boron stabilization of ribose, (3) tungsten catalysts and (4) soluble polyphosphates led to precursor versions of RNA and then via ATP to DNA. It is important to note that fumarolic fluids can contain adequate concentrations of boron, tungsten, and soluble polyphosphates as well as phospholipids as micelles which can enlarge to vesicles, the latter vital as “reaction chambers”. Equally important are biofilms of iron sulfide to which polar organic molecules could attach and receive energy.

The physical attributes of fumaroles as non-dispersed systems provide a serial or fluctuating change in pH, Eh, temperature, clay types and wetness and dryness within distances of meters. Dripping or spatter phenomena can possibly create a version of a polymerase chain reaction by spatter droplets falling onto hot montmorillonite. Here flash evaporation would concentrate enzymes, catalysts and most importantly divalent ions common in fumarolic fluids such as Ca, Mg, Ba and Zn. These ions could neutralize the divalent PO_4_ radicals making up the backbone of double strand RNA or DNA causing the released nucleotide strands to associate. This process would possibly produce exponential replication of the RNA or DNA molecule. Thus, the fumarolic environment is replete with stimuli favoring the origin of protolife especially when combined with the uniquely lunar conditions of lesser gravity and lower surface atmospheric pressures. Lower gravity would produce a deeper nucleation of biofilm bubbles and a slower rise rate promoting higher probabilities of reactivities. Lesser atmospheric pressures would lower boiling points of prebiotic fluids to temperatures more hospitable for protolife.

Searching for protolife on the moon can utilize specific remote sensing instruments some already designed for use on Mars and the outer planets. In addition to spectral reflectance measurements by the Lunar Mineral Mapper, altitude measurements using P-band radar holds promise for imaging through a dust cover over buried fumaroles. Orbiting active but power limited neutron sensors could analyze a 2.22 gamma backscatter specific to H-bearing sublimates. Surface analyses could benefit from the already designed 10 m standoff laser ablation and Raman spectrometers, the latter being well suited for analysis of fumarolic organic compounds and sublimates. An important altitude or surface probe would be a nuclear spectrometer to detect elements of high neutron capture cross section such as boron and chlorine in possible brines. Finally, for heavy or high power sensors such as differential absorption lidar, their emplacement at appropriate levels on a lunar space elevator might be considered.

In overview, the implications of lunar protolife are profound. The exploration of space has the search for life as one of its main goals. Some of the emphasis of the Mars program deals with finding evidence for life in volcanic vents. Witness the discovery of what appear to be ancient hot springs in the Vernal Crater (Arabia Terra) on Mars [145]. It is logical to search for lunar protolife in fumarolic environments where the building blocks of life may have been present. The thermodynamic potential is there [146]. Even if only amino acids are found in volcanic vents, questions of chirality may be answered. If only racemic amino acids are found on the moon, what implications would this have on our understanding of terrestrial chirality? Protolife in fumarolic vents on the moon would relate to the origin of life on earth. Possibly some terrestrial models (submarine, coastal oceanic, subterranean) would need to be re-examined. Likewise, panspermia would be questioned. On a more provincial scale, the origin of lunar craters may require some revision. Protolife in the breached central mountain in Copernicus would suggest an endogenic origin of this crater. Finally, lunar protolife would intensify exploration for life not only on Mars, Titan, Europa and Enceladus where evidence for water is present, but also for the emerging field of exoplanets.

## Figures and Tables

**Table 1.** Products produced in spark discharge experiments using CH_4_, NH_4_, H_2_O and H_2_ (Miller [33] and others). Formic acid is the most abundant product.

Yields from spark discharge experiments using CH_4_, NH_4_, H_2_O, and H_2_	
**compound**	**percent yield**
Glycine	2.1
Glycolic acid	1.9
Sarcosine	0.25
Alanine	1.7
Lactic acid	1.6
*N*-Methyl alanine	0.07
α-Amino-n-butyric acid	0.34
α-Aminoisobutyric acid	0.007
α-Hydroxybutric acid	0.37
β-Alanine	0.76
Succinic acid	0.27
Aspartic acid	0.024
Glutamic acid	0.051
Iminodiacetic acid	0.37
Iminoaceticpropionic acid	0.13
Formic acid	4.0
Acetic acid	0.51
Propionic acid	0.66
Urea	0.034
*N*-Methyl urea	0.051

**Table 2.** The Stimulus Concept.

**The Stimulus Concept for the Origin Of Protolife in Fumaroles**
A. Abrupt Environmental Changes Hot to cold over meter distances and hot-cold interfaces [111] Intermittent sunlight (on polar crater floors) Freeze-thaw phenomena Wet-dry cycles pH changes Redox potential Conductivity changes Vapor pressure changes with temperature Flash evaporation of droplets containing pre-biotic compounds falling onto hot fumarolic clay, enhancing concentrations Variations in clay types
B. Catalysts Possible proflavine (PAH) for nucleotide assembly into RNA Tungsten for critical tungsto-enzymes Homoionic montmorillonite [116]
C. Electrical Surges Nanocurrents by flow charging processes Nanocurrents by freezing certain fumarolic compounds
D. Concentration of protolife Constituents Versus Oceanic Environments Inorganic Organic (including PAHs) Reflux mechanisms
E. Availability of Multiple Templates and Cages Clay (montmorillonite, kaolinite, etc.) Zeolites Green rust
F. Fluctuating Hydrothermal Environments [112]
G. Iron Sulfide Biofilms as an Energy Source and Platform for Organic Molecule Attachment
H. Lipid Synthesis by Fischer-Tropsch Reactions
I. Zonation of All Parameters within Distances of Meters
J. Lower Gravity and Surface Pressure
K. Tidal Forces
